# Damage Monitoring of Regularly Arrayed Short-Fiber-Reinforced Composite Laminates under Tensile Load Based on Acoustic Emission Technology

**DOI:** 10.3390/polym16070890

**Published:** 2024-03-24

**Authors:** Hongda Cai, Wenlong Lu, Jingxuan Ma, Yinyuan Huang, Junfeng Hu

**Affiliations:** 1 School of Mechanical and Power Engineering, Nanjing Tech University, Nanjing 211800, China; 202121106037@njtech.edu.cn (H.C.); 202261207063@njtech.edu.cn (J.M.); 202161107031@njtech.edu.cn (Y.H.); 2 School of Energy Science and Engineering, Nanjing Tech University, Nanjing 211800, China; 202261208061@njtech.edu.cn; 3 Suzhou Yihe Yongli New Energy Co., Ltd., Suzhou 215400, China; 4 Jiangsu Olymspan Thermal Energy Equipment Co., Ltd., Changzhou 213101, China

**Keywords:** unidirectionally arrayed chopped strands (UACS), acoustic emission technology, tensile test, finite element simulation

## Abstract

Carbon-fiber-reinforced polymer (CFRP) composites are widely used in lightweight structures because of their high specific strength, specific modulus, and low coefficient of thermal expansion. Additionally, the unidirectionally arrayed chopped strand (UACS) laminates have excellent mechanical properties and flowability, making them suitable for fabricating structures with complex geometry. In this paper, the damage process of UACS quasi-isotropic laminates under tensile load was tested using acoustic emission detection technology. The mechanical properties and damage failure mechanism of UACS laminates were studied combined with finite element calculation. By comparing and analyzing the characteristic parameters of acoustic emission signals such as amplitude, relative energy, and impact event, it is found that acoustic emission behavior can accurately describe the damage evolution of specimens during loading. The results show that the high-amplitude signals representing fiber fracture in continuous fiber laminates are concentrated in the last 41%, while in UACS laminates they are concentrated in the last 30%. In UACS laminates, more of the damage is caused by matrix cracks and delamination with medium- and low-amplitude signals, which indicates that UACS laminates have a good suppression effect on damage propagation. The stress–strain curves obtained from finite element analysis agree well with the experiment results, showing the same damage sequence, which confirms that the model described in this research is reliable.

## 1. Introduction

Carbon-fiber-reinforced polymer (CFRP) composites have excellent mechanical properties, such as a high specific modulus, high strength, corrosion resistance, and low thermal expansion coefficient of excellent performance, and show obvious performance advantages compared with traditional metal materials [1]. Therefore, composite materials have been widely applied in various fields including aerospace, new energy, and transportation, among others. In commercial aircraft like the A350 and Boeing 787, these composite materials constitute approximately 52% and 50% of the total structural weight, respectively, consisting of carbon fiber composites, glass fiber composites as well as hybrid composites [2]. In the wind power industry, there is an urgent need for large-scale turbine blades because of the continuous increase in the power generation capacity of single wind turbines. The application of carbon fiber composite materials in critical bearing structures of blades, such as the blade web and main beam cap, can effectively optimize blade weight while enhancing structural stiffness [3]. In the field of transportation, including automobiles and rail vehicles, it could promote the lightweight design of traffic vehicles, improve their mobility and safety, and efficiently reduce fuel consumption and emission by replacing traditional metal materials with CFRP composites, to make technical reserves for the development of the next generation high-speed transportation [4,5]. It has been proven that CFRP laminates can be designed to meet the structural requirements and mechanical properties of components according to specific types of loads [6].

There are inevitably structures with complex geometry in buildings. Therefore, it is required that carbon fiber composites have good formability to realize material substitution or use as reinforcement structures [7]. However, conventional CFRP composites have certain limitations in manufacturing intricate structural components due to their poor flowability, and it is easy to generate resin-rich regions at sites with a sharp change in geometry [8]. The existence of such defects leads to the accumulation of complex stress concentration, which will influence structural performance and cause safety risks to the structures [9]. Short-fiber-reinforced polymer (SFRP) composites exhibit better energy absorption characteristics and progressive failure characteristics compared with traditional continuous carbon fiber [10]. The excellent flow performance of SFRP can ensure a more uniform distribution of discontinuous fibers and matrix during the fabrication of structures with complex geometries to avoid the stress concentration [11]. Sheet molding compound (SMC) has gained significant attention and widespread application in the field of SFRP due to their exceptional flow performance and cost-effectiveness. However, the strength of SMC decreases significantly compared to conventional continuous fiber composites [12,13]. The development of a kind of SFRP with a unidirectional fiber distribution was attempted by certain researchers in an effort to enhance the mechanical characteristics of SFRP. Taketa, a scholar from Tohoku University in Japan, introduced a new type of short-fiber-reinforced polymer composite; that is, unidirectionally arrayed chopped strands (UACSs) were introduced into the traditional continuous CFRP prepregs and then stacked according to the designed layering order [10]. UACS laminates exhibit superior mechanical properties compared to previous SFRP while maintaining excellent fiber flowability. The slits in the initial design of UACS laminates were distributed vertically with the fiber direction. However, this type of slit significantly reduced the in-plane strength of CFRP laminates, making them prone to extensive delamination over large areas at low load levels due to damage along the slits. The subsequent UACS design incorporated continuous slits with a small angle, resulting in a reduction in the normal stress experienced by the initial UACS laminates under tensile load and the enhancement of its mechanical properties to some extent. Experimental results indicate that in laminates where all the slits are continuously penetrated through the prepreg, delamination occurs rapidly along the slit direction once it occurs. This results in a loss of bearing capacity and eventual failure of the laminates. To improve this problem, Wang et al. proposed a novel design featuring a discontinuous slit structure. Currently, small-angle staggered and bi-angled slits have been identified as exhibiting the most favorable behavior. Experimental results demonstrated that the tensile strength of these two types of UACS laminates reached 64% and 67%, respectively, compared to traditional CFRP laminates [14,15]. In addition, compression experiments showed that the energy absorption performance of the novel UACS laminates is nearly 49% higher than that of the traditional CFRP laminates [16].

However, detecting failure patterns accurately in UACS laminates was complicated due to a large number of discontinuous slit structures. At present, the common nondestructive testing methods for fiber composite materials include X-ray testing, ultrasonic testing, infrared thermal wave imaging testing, acoustic emission testing, microwave testing, and so on [17,18]. Among them, acoustic emission (AE) is the release of transient elastic waves due to rapid energy release in a localized part of a material [19]. The transducer converts the surface vibration caused by the AE wave transmitted to the surface of the material and measures the change in the output electrical signal to realize the indirect estimation of the AE wave, the monitoring of damage and the evaluation of structural integrity [20]. In the damage detection of CFRP laminates, AE technology could distinguish fiber fracture, matrix damage, delamination, and debonding according to signal characteristics. High energy fiber brittle fracture is often accompanied by high amplitude and energy signals. In contrast, the signals generated by matrix cracking usually have lower amplitude and energy. The analysis of these signals could accurately monitor the health of the composite laminates and ensure their safety. P. Wysmulski et al. proposed a new method for determining initial failure inside CFRP columns based on AE technology [21]. By analyzing the amplitude of the AE signal, corresponding to the load time curve, the load where the initial damage occurs can be effectively obtained. L. B. Andraju et al. used AE and digital image correlation (DIC) techniques to study the internal damage of CFRP laminates under tensile load, including matrix cracking, debonding, and fiber fracture [22]. According to the microscopic appearance of the damage, the AE signals are assigned and identified, and the frequency range of the AE signals for different types of damage is given. The results show that AE is ideal in understanding the damage mechanism and failure mechanism of CFRP composites. Baker et al. [23] used energy and peak frequency to detect the crack initiation point and crack propagation of CFRP laminates. When the stress increases obviously, the energy and peak frequency are positively correlated with the crack density. The results show that the peak frequency alone could not describe the initial damage of matrix cracking, but the combination of peak frequency and AE energy can effectively detect the crack. Cheng et al. prepared CFRP laminates with interlayer toughening of carbon nanotubes (CNTs) and studied their damage modes using a double cantilever beam (DCB) test [24]. The results show that the AE signals obtained are obviously clustered, and the damage can be effectively separated by peak frequency. In addition, the difference in damage evolution between fabric and unidirectional laminates is explained from the perspective of energy, which can be reflected in AE signals. E. Morokov et al. [25] monitored the three-point bending experiment of orthogonal carbon fiber laminates by AE and pointed out that the AE events and energy levels are related to the decline and slope of the load curve. Li et al. [26] used AE to monitor the damage to honeycomb sandwich cylinders, and the damage to complex structures can still be accurately monitored by resolving the peak frequency of AE signals. Liu and Ren et al. combined AE technology with deep learning to further improve the accuracy of damage identification, showing great potential in damage monitoring [27,28].

The AE technique is an effective tool for detecting flaws and improving research validity. Zhou et al. studied the compressive damage buckling behavior of composite materials based on the AE signal characteristics, micro-displacement field, and strain field, and found that the parameters of AE showed a strong correlation with the damage progression of composite specimens [29]. Zhang et al. studied the damage evolution mechanism of CFRP composites during the tensile process. The surface deformation and internal damage evolution of the materials can be reflected by the real-time tensile displacement field and AE characteristic parameters, respectively, and the results show that the failure mode of the specimens is a brittle fracture [30]. Wildemann et al. [31] proposed a method to study the damage accumulation process of composite materials with the application of digital AE, including tensile mechanical tests on flat open-hole specimens. According to the AE data, the location diagram, peak amplitude, and frequency distribution diagram of AE signals were drawn. Although AE technology has been used for detecting the internal damage of carbon fiber composite materials, for UACS laminates with more complex structures and relatively weak mechanical properties, whether AE can accurately describe the internal damage mechanism is still worthy of further study, which is crucial to detect the damage process of UACS laminates and explain the damage evolution.

Therefore, in this paper, AE is used to study the tensile properties of UACS laminates with slits and continuous fiber laminates, so as to detect the damage of UACS laminates with degraded strength and study the various damage modes and complex failure mechanism of UACS laminates with slits. The damage mechanisms of laminates during different loading stages were determined by the AE characteristics. Different signal characteristics, such as the amplitude, cumulative event, and cumulative energy, are used to distinguish the damage modes of UACS laminates and continuous fiber laminates. In addition, the finite element models of UACS laminates and continuous fiber laminates were established, respectively, to predict the damage evolution process of internal structure under tensile load, including delamination and slit patterns. Finally, the accuracy of the numerical model is verified by comparing the damage appearance observed in the experiment. The final tensile fracture pattern of UACS laminates is analyzed according to various damage modes in UACS laminates.

## 2. Analysis Procedure

### 2.1. Materials

**Figure 1:**
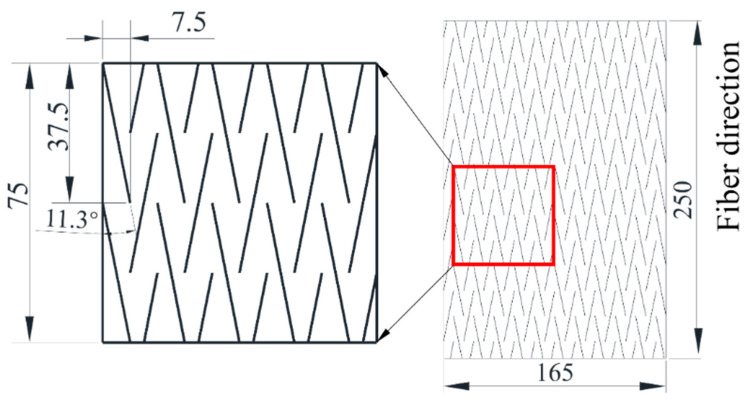
Dimension of prepreg layer for tensile specimens and slits of UACS prepreg.

**Figure 2:**
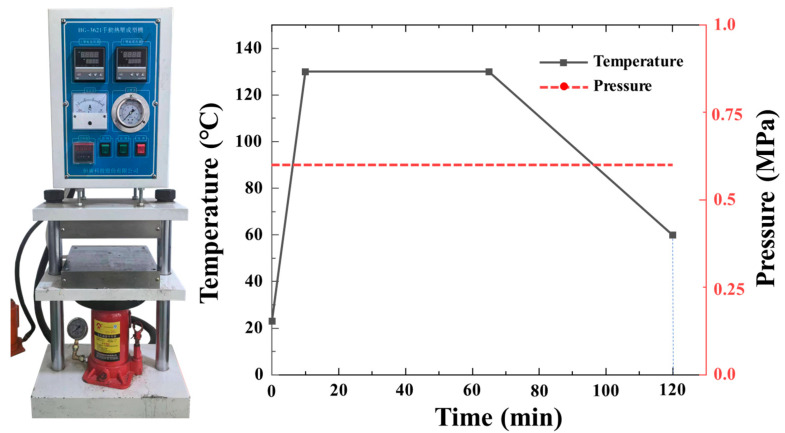
Curing curve of composite laminates.

The CFRP prepreg applied in this study is the standard thickness carbon fiber prepreg T800/7901 (thickness: 0.125 mm) produced by Weihai Guangwei Composite Materials Co., Ltd. (Weihai, China). The mechanical property parameters of the prepreg are listed in Table 1. Prepreg layers and bi-angled slits are cut by a CNC cutting machine to ensure dimensional accuracy. The cutting size of the prepreg is 250 × 165 mm. The schematic diagram of UACS carbon fiber prepreg is shown in Figure 1. The prepregs are stacked in sequence according to the laying sequence of quasi-isotropic laminates [45°/0°/−45°/90°]s, then placed in the mold. According to whether the carbon fiber layer is continuous, it is divided into continuous specimens T-C-8 and UACS specimens T-U-8, where T stands for tensile specimen, C stands for continuous carbon fiber, U stands for UACS laminate, and 8 stands for the number of carbon fiber layers. In this study, the laminates are fabricated using hot pressing. During this process, the pressure and temperature parameters of the thermal press are adjusted, with a pressing time of 60 min, a temperature of 130 °C, and a molding pressure of 0.6 MPa. This ensures a stable hot pressing curing process, as shown in Figure 2, to guarantee the quality of the laminates. Once the curing process is complete, the heating is turned off while the pressure is kept constant. The laminate is cooled naturally to below 50 °C before pressure relief.

### 2.2. Tensile Strength Test and Acoustic Emission Detection

**Figure 3:**
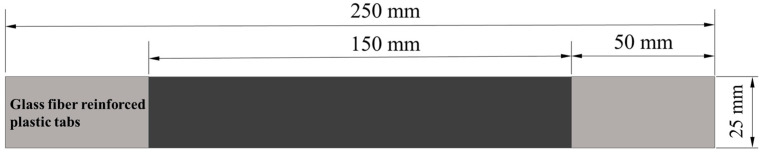
Dimensions of tensile specimen.

According to ASTM D3039M [32], the size of the tensile specimen is 250 × 25 mm; specimens are water-cut from the laminate to meet size requirements. The size of the tensile specimens is shown in Figure 3, the gauge distance is 150 mm, and a glass fiber reinforcement sheet has been attached to both ends of the specimens, the size of which is 50 × 25 mm.

**Figure 4:**
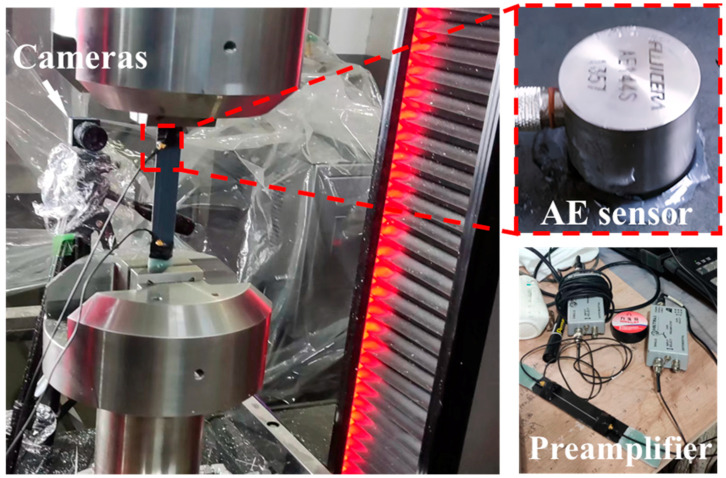
Experimental setup of tensile test including DIC and AE equipment.

The tensile test refers to the experimental standard ASTM D3039M, and the configuration of the experiment is illustrated in Figure 4, including a universal testing machine (LE5305, Lishi Shanghai Scientific Instrument Co., Ltd., Shanghai, China). The tensile speed is set at 1 mm/min, and the sampling frequency is 4 Hz. The speckle images were taken with an industrial camera (acA2500, Basler, Ahrensburg, Germany, with a frequency of 60 Hz), and the tensile strain field was calculated using VIC-2D V6 software. In addition, the strain data measured by a large-range extensometer (3542-050M-010-ST, Epsilon, Jackson, WY, USA) are compared with the results of the DIC method measurement. Continuous fiber laminates and UACS laminates were tested four times to ensure the accuracy of the test. The Mistras Micro-II digital AE system (Princeton Junction, NJ, USA) uses AEwin’s PAC PCI-2 software for AE testing, with hardware components primarily consisting of AE sensors, preamplifiers, cables, and other related components. The considered quasi-static tensile test is stable and produces less noise interference. The two AE sensors were connected to the specimen surface using Vaseline as a coupling agent and secured with tape at a distance of about 120 mm. It can greatly reduce the signal attenuation on both surfaces and ensure better signal transmission performance between the specimen and the AE sensor. The speed of sound in each specimen is determined by a pencil break lead test prior to the AE test. Characteristic parameters such as time, impact event, amplitude, and relative energy are extracted from the AE signals collected by the AE system, and the corresponding coordinate images are sorted. Since AE signals are non-stationary signals, useful signals are easily interfered with. In order to ensure the validity of the analysis results, the pencil breaking test was carried out. Breaking the pencil simulates small damage and produces AE signals, and filtering out the signal below the break lead test threshold can effectively separate the noise. This method is frequently used for calibrating AE sensors because of its excellent repeatability, easy implementation, and signal stability.

### 2.3. Finite Element Analysis Model

**Figure 5:**
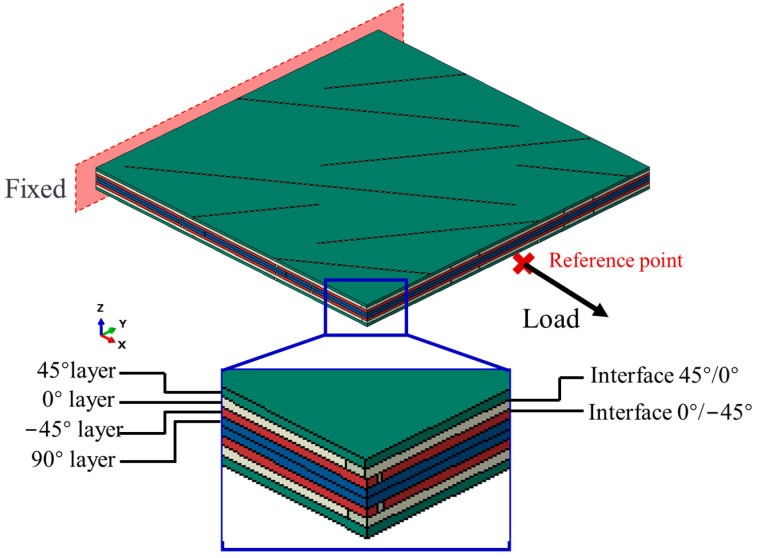
Finite element model of UACS laminates.

To simulate the tensile process of continuous laminate, a full-size finite element analysis model is created using ABAQUS 2021 software. Figure 5 shows the schematic diagram of the finite element model of the UACS specimen. The numerical model of UACS laminates divides each layer into two sections, each of which is assigned a certain material property. The rest region outside the slits is referred to as anisotropic CFRP prepreg, and slits are classified as resin as they are filled with resin during the curing process. The material direction of each layer model is consistent with the test, to establish a quasi-isotropic UACS model, and the model size is 25 mm × 25 mm. The thickness direction has been configured to consist of 8 layers, the stacking sequence is [45°/0°/−45°/90°]s, and each layer is 0.13 mm thick. Material properties of the composite and cohesive elements are defined in the material manager, and the properties of epoxy resin are listed in Table 2. The mesh size is approximately 0.5 mm globally, taking into account the geometry of the prepreg and the slits; this size can meet the calculation accuracy and ensure good efficiency. For the capture of complex interactions within the UACS laminates, the extensively accepted Hashin’s failure criterion was employed to distinguish and determine various damage modes, which is also in line with the complexity of damage influence on the material properties. The Hashin criterion is expressed as follows:

Fiber tensile failure ($\sigma_{11}\geq 0$)

(1) $$r_{\mathrm{ft}}={\left(\frac{\sigma_{11}}{X_{T}}\right)}^{2}+{\left(\frac{\sigma_{12}}{S_{12}}\right)}^{2}\geq 1$$

Fiber compressive failure ($\sigma_{11}<0$)

(2) $$r_{\mathrm{fc}}={\left(\frac{\sigma_{11}}{X_{C}}\right)}^{2}\geq 1$$

Matrix tensile failure ($\sigma_{22}\geq 0$)

(3) $$r_{\mathrm{mt}}={\left(\frac{\sigma_{22}}{Y_{T}}\right)}^{2}+{\left(\frac{\sigma_{12}}{S_{12}}\right)}^{2}\geq 1$$

Matrix compressive failure ($\sigma_{22}<0$)

(4) $$r_{\mathrm{mc}}={\left(\frac{\sigma_{22}}{2S_{21}}\right)}^{2}+\frac{\sigma_{22}}{Y_{C}}\left[{\left(\frac{Y_{C}}{2S_{21}}\right)}^{2}-1\right]+{\left(\frac{\sigma_{12}}{S_{12}}\right)}^{2}\geq 1$$

where $r_{\mathrm{ft}}$, $r_{\mathrm{fc}}$, $r_{\mathrm{mt}}$, and $r_{\mathrm{mc}}$ are the initial damage satisfaction degrees corresponding to the four failure modes. $\sigma_{\mathrm{ij}}$ (i, j = 1,2) defines the equivalent stress tensor. $X_{T}$ and $X_{C}$ signify the tensile and compressive strengths of normal direction. $Y_{T}$ and $Y_{C}$ are the tensile and compressive strengths in the transverse direction. $S_{12}$ are the shear strengths.

The quadratic failure criterion and B-K criterion predict the damage propagation of interlaminar delamination and mixed-mode loading failure behavior, respectively. In composite laminates, the interface between adjacent layers is described as zero-thickness cohesive elements with a bilinear traction–separation relationship, as shown by Equations (5) and (6):

(5) $$\;{\left\{\frac{t_{n}}{t_{n}^{o}}\right\}}^{2}+{\left\{\frac{t_{s}}{t_{s}^{o}}\right\}}^{2}+{\left\{\frac{t_{t}}{t_{t}^{o}}\right\}}^{2}=1$$

(6) $$G^{C}=G_{n}^{C}+\left(G_{s}^{C}-G_{n}^{C}\right){\left\{\frac{G_{s}}{G_{t}}\right\}}^{\eta }$$

The properties of cohesive elements are shown in Table 3. The element type of carbon fiber is set as the universal continuous shell with a second-order continuum element with eight nodes, namely SC8R elements, and the element type of cohesive element is set as COH3D8, which is a continuum element with hourglass control, used in three-dimensional analysis, also with eight nodes. In this study, the structured grid is generated by scanning. The total number of elements is 60,000 with 32,000 SC8R and 28,000 COH3D8 elements. The number of nodes is 66,976. In the loading process, as shown in Figure 5, the degree of freedom of one side is restricted so that it is completely fixed, the node of the opposite side is coupled to the reference point, and then the tensile load is applied to the reference point in a smooth analysis step until the laminate fails.

## 3. Results and Discussions

### 3.1. Results of Tensile Tests

**Figure 6:**
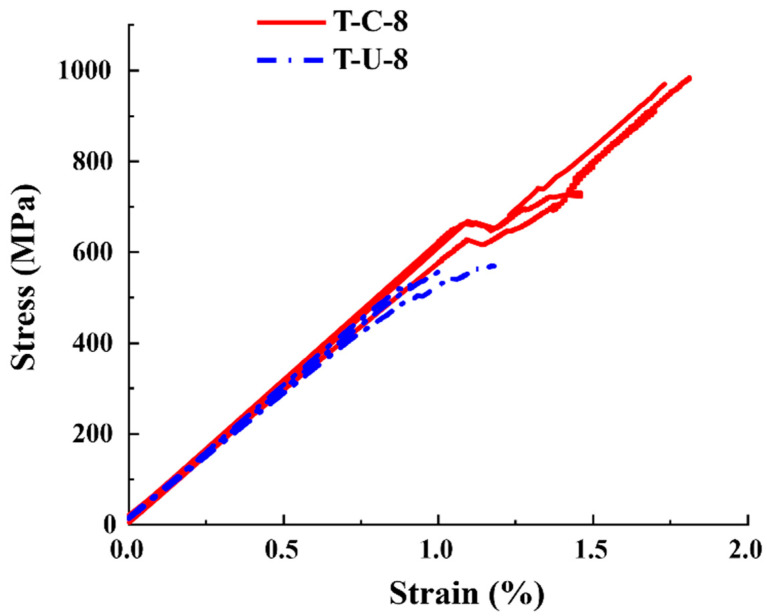
Tensile stress–strain curves of specimens of continuous fiber laminates and UACS laminates.

Figure 6 shows the tensile stress–strain results of continuous fibers and UACS specimens under quasi-static tensile load. The tensile strength and elastic modulus of T-C-8 and T-U-8 specimens are 951.66 MPa, 60.282 GPa, and 551.43 MPa, 59.241 GPa, respectively. Compared with specimen T-C-8, the average tensile strength and average elastic modulus of T-U-8 specimens decreased by 42.06% and 1.73%, respectively. This is because the slits in UACS laminates will significantly weaken the strength of the in-plane quasi-isotropic laminates while having little effect on their tensile modulus. All specimens show the linear elastic region in the early part of the curve, and the modulus of both specimens is almost the same in the linear stage. Until the late loading period, specimens T-C-8 and T-U-8 have slight progressive damage. It is worth noting that almost all T-C-8 specimens showed nonlinear load curve fluctuations at a strain of 1.1%, which continued to rise after a slight load drop, finally resulting in brittle failure at 1.9% strain. The stress–strain curves of UACS specimens show an obvious nonlinear response at the late loading stage, which indicates that the slits inside UACS have a positive effect on the progressive damage of the laminates. During the tensile process, the interface dislocation along the slits at different layers of UACS laminates will lead to progressive delamination propagation (nonlinear response on the stress–strain curve).

**Figure 7:**
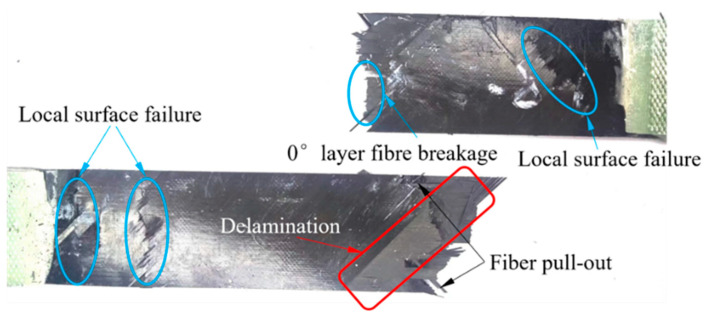
Typical image of the final tensile fracture pattern of continuous carbon fiber laminates.

**Figure 8:**
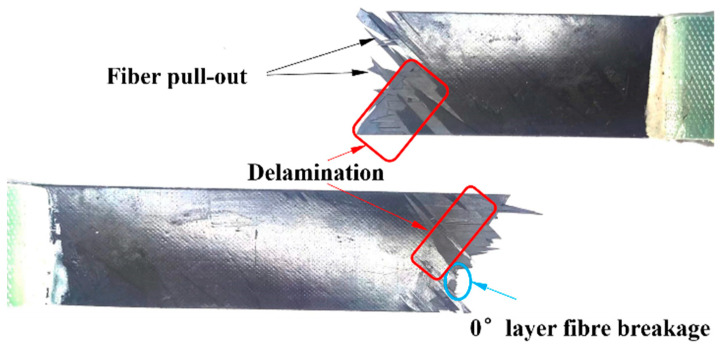
Typical image of the final tensile fracture pattern of UACS laminates.

Figure 7 and Figure 8 display the typical fracture morphology of continuous carbon fiber laminates and UACS laminates after tensile failure, respectively. It is evident from Figure 7 that the continuous fiber specimen exhibited significant damage in several regions, mainly near the middle and end of the gauge length. The damage included delamination primarily at the 45° interlayer interface and local fiber fracture. However, there was only one final fracture. This can explain the load fluctuation at a strain of 1.1% in Figure 6. During the bearing process of the specimen, the local fiber fracture and delamination in the 45° surface layer resulted in an instantaneous decrease in the bearing property of the specimen, but the fiber in the 0° layer did not fracture and could continue to bear the load. It can be seen from the specimen fracture surface that a large area of delamination occurred between the 0° and 45° layers, and a large number of fiber fractures occurred in the 0° layer; eventually, this leads to a decrease in the load bearing capacity of the laminates.

However, it was observed that the UACS laminates experienced some nonlinear behavior before they ultimately failed during the experiment. In UACS laminates, delamination propagation is often concentrated at weak interfaces of slits and expand to the other side of the slits. Under external load, resin deformation in the slits promotes fiber flow on both sides, which means that UACS have a progressive damage process different from continuous fiber laminates. Through a comparison between Figure 7 and Figure 8, it was observable that the fracture of continuous fiber specimens has continuous large-area delamination, while the delamination degree of UACS laminates is significantly smaller than that of continuous fiber specimens and shows more obvious dispersion and a more pronounced fiber pull-out phenomenon.

### 3.2. Results of Acoustic Emission Detection

**Figure 9:**
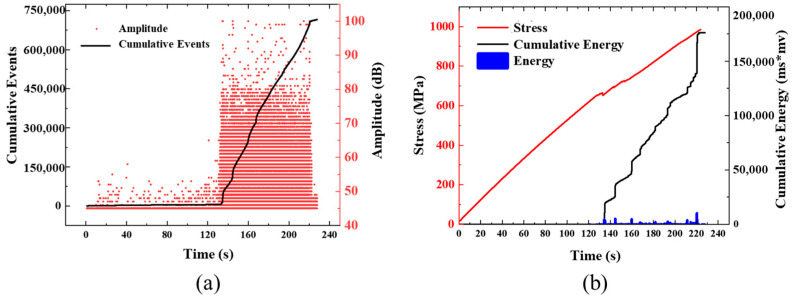
Typical acoustic emission signal of continuous fiber specimens under tensile load: (**a**) amplitude and accumulated events; (**b**) accumulated energy.

**Figure 10:**
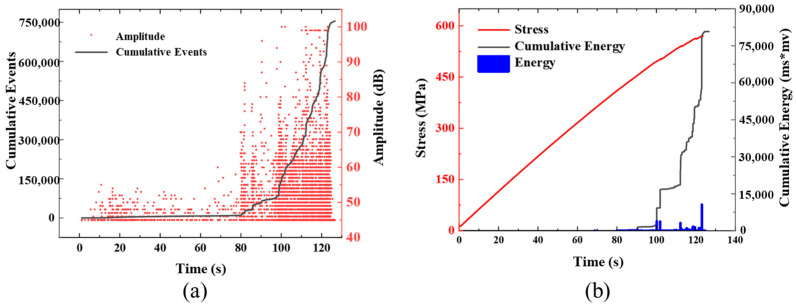
Typical acoustic emission signal of UACS specimens under tensile load: (**a**) amplitude and accumulated events; (**b**) accumulated energy.

Due to the test’s high repeatability, a single group of specimens was selected for further analysis. Figure 9 and Figure 10 show the history curves of AE amplitude-impact cumulation-time, and load-cumulation energy-time of specimen T-C-8 and T-U-8, respectively, during the tensile test. For the specimens of T-C-8, the impact event increased slowly in the first 120 s, and the detected AE signal amplitudes were all between 45 and 60 dB. Until 130 s, the cumulative impact event reached 5398, accounting for only 0.75% of the total impact event. The cumulative relative energy reached 19 ms*mv, which was negligible compared with the total cumulative energy, indicating that no obvious damage occurred in the specimen during this period. From 130 s to 220 s, the cumulative count of impacts increased significantly at an approximate linear rate, with a large number of AE signals of medium and high amplitude appearing simultaneously. Impact events with relative energies exceeding 10,000 ms*mv also began to appear. The main failure modes at this stage are interlayer delamination and fiber/matrix debonding. From 220 s to 222 s, the cumulative relative energy increased by 36,943 ms*mv in less than two seconds, which indicates that the specimen suddenly dropped a load at the last moment of loading, namely, the complete fracture. For specimen T-U-8, the AE signals detected at the initial 80 s of loading are similar to those detected in specimen T-C-8, the cumulative impact event and cumulative relative energy grow very slowly, and the growth slope tends to a horizontal state. In 80 s, the cumulative impact event reached 1042, accounting for 1.38% of the total impact event, and the cumulative relative energy reached 25 ms*mv. From 80 s to 123 s, the cumulative impact event and relative energy increased rapidly, in which the cumulative impact event increased exponentially, and high-amplitude and high-energy AE signals began to appear. Compared with Figure 9 and Figure 10, it was observed that the impact events of the T-U-8 specimens were earlier than the T-C-8 specimens. This is due to the presence of slits that weaken the strength of the laminate, causing the specimens to fail at a relatively low load. Meanwhile, the cumulative impact event and relative energy of specimen T-U-8 are much smaller than that of specimen T-C-8, which is mainly due to the smaller failure strain of T-U-8 and fewer cumulative impact events caused by premature failure. In addition, it could be found that the high-decibel impact events of the continuous fiber specimens are concentrated in the last 41% of the tensile test, while the UACS specimens are in the last 30%, and the overall decibel value level of the impact event is significantly lower, indicating that there is more matrix and delamination damage. Therefore, it could be concluded that although the failure strain of the UACS specimens is lower than that of the continuous fiber, the existence of the slit does inhibit the damage expansion to a certain extent and delay the final failure.

### 3.3. Results of Finite Element Analysis

**Figure 11:**
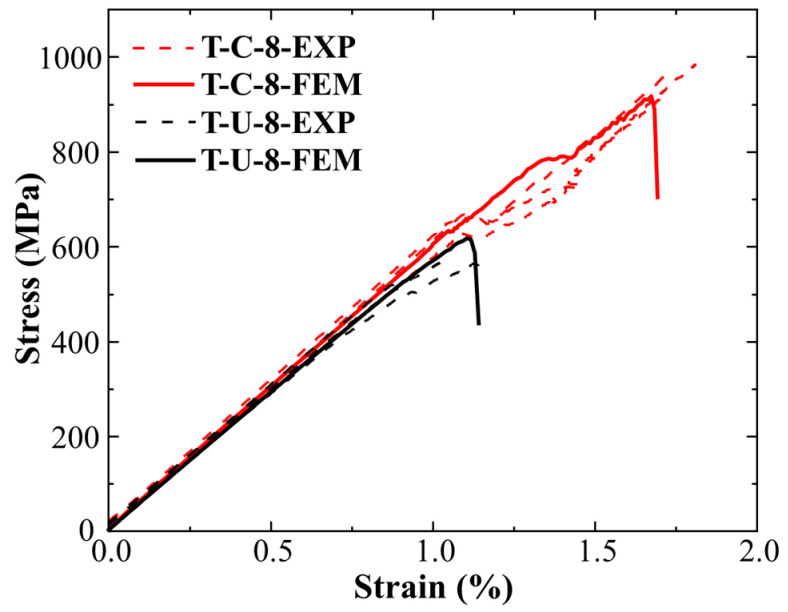
Experimental measurement and comparison of simulated stress–strain curves.

Figure 11 shows a comparison between the stress–strain curves of specimen T-C-8 and T-U-8 obtained from finite element analysis and the experimental results. It is evident that the stress–strain curves show a consistent variation trend between finite element simulation and experiment. The numerical simulation results of strength and modulus of continuous fiber specimens T-C-8 are 917.3 MPa and 61.498 GPa, respectively, which indicate a 3.58% lower strength and a 2.02% higher modulus than the experimental results. Meanwhile, the simulation also captures the nonlinear behavior before the failure. The simulation results of the strength and modulus of UACS laminates are 594.8 MPa and 60.452 GPa, respectively, which are 7.9% and 2.04% higher than the experimental values, showing the high accuracy of the finite element model in strength analysis.

**Figure 12:**
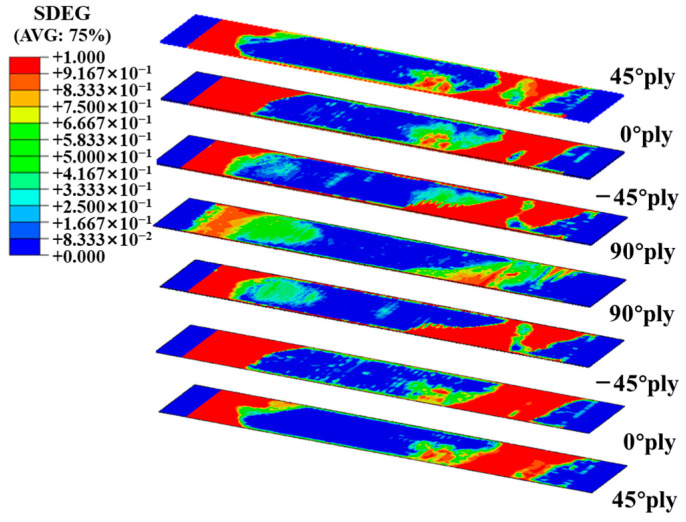
Interface element damage of tensile specimen T-C-8.

Figure 12 shows the interface damage between adjacent layers of continuous fiber laminates. Upon analyzing the results, it appears that the delamination damage is mainly concentrated at both ends and gradually propagates toward the center. All adjacent layers of the specimen exhibited different degrees of delamination damage, which showed a trend of diffusion from the edge to the center, and the delamination area was significantly affected by ±45° layers. Since the layers are ±45° relative to the tensile direction, the shear stress between the layers is the largest, resulting in the material slipping along the layers and delamination damage at the interface of the layers. This was consistent with the multiple failure and delamination morphology observed in Figure 7.

**Figure 13:**
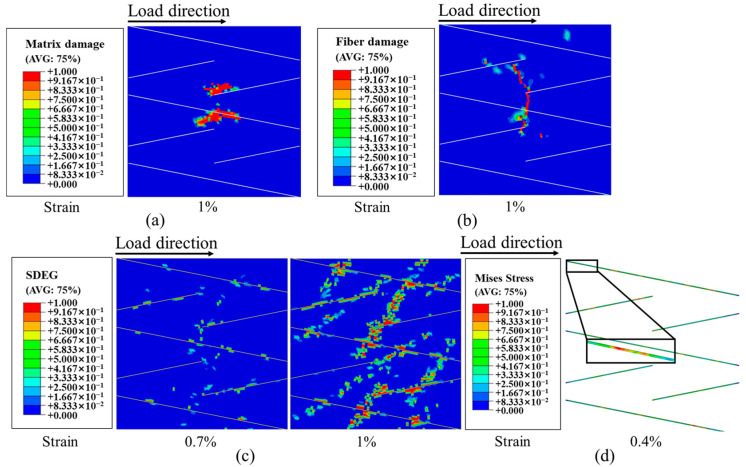
(**a**) Matrix damage and (**b**) fiber damage of 0° layer of UACS laminates before final failure; (**c**) damage development of 0°/−45° interface layer of UACS laminates; (**d**) von Mises stress of slits in 0° layer at the strain of 0.4%.

Figure 13 illustrates different damage processes that occur in UACS laminates when subjected to tensile loading. The damage to the fiber and matrix in the 0° layer before the ultimate failure of UACS laminates is shown in Figure 13a,b. The primary tensile load was borne by the fibers in the 0° layer due to their consistency with the loading direction. Fiber fracture is the main cause of failure in the 0° layer, while other layers primarily experience matrix damage and localized delamination. The delamination of the UACS laminates began at the ends of the slits in the 0°/−45° interface layer when the strain reached 0.7%, as shown in Figure 13c. After that, it spread in the 0° layer’s direction of the slits. Most of the delamination took place between the 0° layer and −45° layer until the laminates experienced complete failure. In the case of UACS laminate, the slits in the 0° layer fail at a strain level of 0.4%, as shown in Figure 13d. The matrix of the 90° layer and ±45° layers experienced progressive damage as the load increased, reaching a tensile strain of 0.5% and 0.7%. It was found that matrix microcracks first appeared at the slits of UACS laminates. Therefore, as shown in Figure 13b,c, delamination is usually concentrated at the weak interface of the slits, and transverse through-delamination is generated with the increase in strain, so a relatively flat fiber fracture shape is easily formed.

The existence of slits leads to the change in stress distribution in the laminate, and the damage is mostly concentrated near the slits, which suppress the propagation of delamination damage. It is important to mention that the position of the matrix damage in the 45° layer shows a strong correlation with the slits present in the adjacent 0° layer. Once the strain reaches 1%, the occurrence of fiber fracture in the 0° layer ultimately leads to failure in UACS laminates.

## 4. Conclusions

In this paper, quasi-isotropic continuous fiber laminates and UACS laminates were prepared, and a quasi-static tensile test was carried out to examine the specimen’s mechanical reaction and failure mechanism; acoustic emission technology was utilized, and the characteristic parameters were used to detect the damage to the specimens; and finite element analysis was conducted to investigate the tensile mechanical properties and damage mechanisms of UACS laminates. The conclusions are as follows:

(1) The tensile strength and elastic modulus of the continuous fiber laminates are 951.66 MPa and 60.282 GPa, and these values are 551.43 MPa and 59.241 GPa for UACS laminates. Slits inhibit the delamination of UACS laminates and cause it to have an obvious nonlinear response before the final failure.
(2) The high-decibel impact events of continuous fiber specimens are concentrated in the last 41% of the tensile test, while that of the UACS specimen is concentrated in the last 30%, and the overall decibel level of impact events is low. This indicates that the damage of UACS laminates is mostly matrix damage and delamination because this damage exhibits lower energy and occurs to a smaller degree.
(3) The errors rates in the strength and modulus of continuous fiber laminates are 3.58% and 2.02%, and those of UACS laminates are 7.9% and 2.04%, respectively. The finite element results show that the continuous fiber laminates experienced extensive delamination damage before final failure affected by ±45°. The failure mode of the 0° layer in UACS laminates is fiber fracture, and the other layers experience matrix damage and local delamination, rather than widespread delamination.

## Figures and Tables

**Table 1:** Mechanical properties of CFRP prepregs.

Parameters	T800/7091(CFRP)
Longitudinal Young’s modulus E_1_ (GPa)	145
Transverse Young’s modulus E_2_ = E_3_ (GPa)	9.2
In-plane shear modulus G_12_ = G_31_ (GPa)	4.5
Poisson’s ratio υ	0.27
Longitudinal tensile strength X_t_ (MPa)	2600
Longitudinal compression strength X_c_ (MPa)	1150
Transverse tensile strength Y_t_ = Z_t_ (MPa)	70
Transverse compression strength Y_c_ = Z_c_ (MPa)	180
Out-of-plane shear strength S_23_ (MPa)	55

**Table 2:** Properties of epoxy resin.

Parameters	Value
Elastic modulus E (GPa)	3
Poisson’s ratio ν	0.35
Strengths in normal direction $t_{n}^{0}$ (MPa)	79
Strengths in first shear direction $t_{s}^{0}$ (MPa)	55
Strengths in second shear direction $t_{t}^{0}$ (MPa)	55

**Table 3:** Properties of cohesive elements.

Kn * (GPa/mm)	Ks = Kt * (GPa/mm)	$t_{n}^{0}$ * (MPa)	$t_{s}^{0}=t_{t}^{0}$ * (MPa)	$G_{n}^{c}$ * (N/mm)	$G_{s}^{c}=G_{t}^{c}$ * (N/mm)	η *
100	100	40	50	0.293	0.631	1.45

* Kn, Ks, and Kt—stiffnesses in the normal direction, the first shear direction, and the second shear direction, respectively. $t_{n}^{0}$, $t_{s}^{0}$, and $t_{t}^{0}$—strengths of the nominal stress in the normal direction, the first shear direction, and the second shear direction, respectively. $G_{n}^{c}$, $G_{s}^{c}\;$ and $G_{t}^{c}$—separation energies in the normal direction, the first shear direction, and the second shear direction, respectively. η—material coefficient of B-K criterion.

## Data Availability

Data available on request from the authors.
